# Multifunctional Fe_3_O_4_-Au Nanoparticles for the MRI Diagnosis and Potential Treatment of Liver Cancer

**DOI:** 10.3390/nano10091646

**Published:** 2020-08-21

**Authors:** Elena Kozenkova, Kateryna Levada, Maria V. Efremova, Alexander Omelyanchik, Yulia A. Nalench, Anastasiia S. Garanina, Stanislav Pshenichnikov, Dmitry G. Zhukov, Oleg Lunov, Mariia Lunova, Ivan Kozenkov, Claudia Innocenti, Martin Albino, Maxim A. Abakumov, Claudio Sangregorio, Valeria Rodionova

**Affiliations:** 1 Institute of Physics, Mathematics and Information Technology, Immanuel Kant Baltic Federal University, 236008 Kaliningrad, Russia; ekozenkova@kantiana.ru (E.K.); elevada@kantiana.ru (K.L.); asomelyanchik@kantiana.ru (A.O.); spshenichnikov1@kantiana.ru (S.P.); ikozenkov@kantiana.ru (I.K.); vvrodionova@kantiana.ru (V.R.); 2 Laboratory of Biomedical Nanomaterials, National University of Science and Technology “MISiS”, 119049 Moscow, Russia; efremova33@mail.ru (M.V.E.); nalench92@gmail.com (Y.A.N.); anastasiacit@gmail.com (A.S.G.); dgzhukov@fastmail.com (D.G.Z.); abakumov_ma@rsmu.ru (M.A.A.); 3 Department of Chemistry, Lomonosov Moscow State University, 119991 Moscow, Russia; 4 Department of Medical Nanobiotechnology, Russian National Research Medical University, 117997 Moscow, Russia; 5 Institute of Physics of the Czech Academy of Sciences, 18200 Prague, Czech Republic; lunov@fzu.cz; 6 Institute for Clinical & Experimental Medicine (IKEM), 14021 Prague, Czech Republic; mariia.lunova@googlemail.com; 7 Institute of Chemistry of Organometallic Compounds – C.N.R., 50019 Sesto Fiorentino, Italy; claudia.innocenti@unifi.it; 8 INSTM and Dept. of Chemistry, University of Florence, 50019 Sesto Fiorentino, Italy; martin.albino@unifi.it

**Keywords:** magnetic-plasmonic nanoparticles, nano-heterostructures, MRI contrast agent, liver cancer, theranostic

## Abstract

Heterodimeric nanoparticles comprising materials with different functionalities are of great interest for fundamental research and biomedical/industrial applications. In this work, Fe_3_O_4_-Au nano-heterostructures were synthesized by a one-step thermal decomposition method. The hybrid nanoparticles comprise a highly crystalline 12 nm magnetite octahedron decorated with a single noble metal sphere of 6 nm diameter. Detailed analysis of the nanoparticles was performed by UV-visible spectroscopy, magnetometry, calorimetry and relaxometry studies. The cytotoxic effect of the nanoparticles in the human hepatic cell line Huh7 and PLC/PRF/5-Alexander was also assessed. These Fe_3_O_4_-Au bifunctional nanoparticles showed no significant cytotoxicity in these two cell lines. The nanoparticles showed a good theranostic potential for liver cancer treatment, since the r_2_ relaxivity (166.5 mM^−1^·s^−1^ and 99.5 mM^−1^·s^−1^ in water and HepG2 cells, respectively) is higher than the corresponding values for commercial T_2_ contrast agents and the Specific Absorption Rate (SAR) value obtained (227 W/g_Fe_) is enough to make them suitable as heat mediators for Magnetic Fluid Hyperthermia. The gold counterpart can further allow the conjugation with different biomolecules and the optical sensing.

## 1. Introduction

Heterodimeric nanoparticles (NPs) comprising a magnetic and a metal noble moiety, assembled in different architectures, have attracted considerable attention in the recent past. In fact, the combination of the unique physical properties, which these materials bear at the nanoscale, offers the opportunity to realize complex multifunctional nanoplatforms potentially exploitable in a large variety of technologies, biomedicine undoubtedly being the most prominent [1,2,3,4,5]. In this field, indeed, magnetic-plasmonic hybrid NPs have been proposed for application for drug delivery [1,6], the photothermal depletion of cancerous cells [7], magnetic hyperthermia [8], protein separation [9], cell imaging applications [10], gene transfection [11], magnetic resonance imaging (MRI) [12,13], and biosensing [11,14]. Most of the research has been focused on magnetite/gold hybrid nanostructures, since these two components are endowed of good magnetic and optical properties, respectively, and they are generally assumed to be biocompatible [15,16]. It is also important to highlight that for application in the human body NPs must be properly functionalized to be dispersed in water or other water-based fluids (blood) [17,18].

The capability of magnetic NPs of being manipulated by an external magnetic field and of enhancing the contrast in MRI has been exploited for a long time [4,16,19]. Besides the employment in MRI diagnostics, the application of magnetic NPs has been also envisaged as heat mediators in magnetic fluid hyperthermia (MFH) [20,21,22,23,24], making them the most relevant examples of “theranostic” tools for cancer treatment. In MFH, magnetic NPs are injected or delivered by chemical or magnetic targeting in the tumor tissue and excited by an alternating magnetic field. The energy of the field, absorbed by the magnetic NPs, is released in the environmental tissues, inducing apoptosis or ablation of the cells, depending on the local temperature achieved. Due to its higher selectivity and lower side effects with respect to conventional cancer treatment (Radio/Chemo therapies), the MFH is nowadays considered one of most appealing protocols to kill cancerous cells by a local temperature increase (around 41–46 °C for 30 min). On the other hand, gold NPs of different sizes and shapes have been investigated as photo-thermal/photo-acoustic agents for tumor diagnosis and therapy [25,26], computed tomography (CT) contrast agents [27,28], biosensors [29,30] and catalysts [31,32]. Combining the optical and magnetic properties of gold and magnetite in hybrid nanostructures can thus enhance their functionality and pave the way towards multimodal applications [33]. As an example, the strong contrast in MRI and X-ray CT suggests that the magneto-plasmonic NPs have a large potential as efficient multi-modal imaging probes [34]. Such an approach can be particularly promising for the successful early diagnosis of human hepatocellular carcinoma (HCC), since both gold and magnetite NPs, separately, have proven to be effective contrast agents for the two complementary diagnostic modalities for liver cancer [35,36].

The combination in a common platform of these two highly biocompatible moieties bearing different potential functionalities, which can be tuned toward specific applications by an appropriate synthetic strategy, represents a step forward the realization of a multifunctional nanodevice for biomedicine. Moreover, the presence of two distinct surfaces offers the opportunity for the conjugation with different biomolecules, drugs, and fluorescent dyes.

However, despite this high potential, the translation to clinics of inorganic nanostructures is so far limited to a few notable examples, mainly due to the severe limitations imposed by biological barriers in the human body, such as the mononuclear phagocytic system and the renal clearance pathway. A statistical analysis across the last 30 years of literature, indeed, estimated that only a median of 0.7% of the injected dose reaches the target after systemic administration, the amount depending on the size, coating and charge of the NPs [37]. As an example, recently, Efremova et al. [38,39] synthesized fluorescent-labeled Fe_3_O_4_-Au Janus nanostructures and investigated their biodistribution in mice after intravenous administration, finding a prevalent accumulation in the liver. Nevertheless, the same NPs were demonstrated to be a very promising platform for theranostics [38]. Additionally, one has to bear in mind the potential cytotoxicity of NPs [40,41], which has to be assessed carefully.

To exploit these results, we checked their validity starting from a realistic biological model, HCC, which represents the natural, physiological target of the injected NPs. This work is thus focused on the synthesis of Fe_3_O_4_-Au nano-heterostructures comprising a small gold nanosphere coupled to octahedral single crystal of magnetite as theranostic agents for liver cancer treatment. For this purpose, optical, magnetic, relaxometric and hyperthermic properties of the NPs are analyzed together with biocompatibility, toxicity and contrast efficacy in different human hepatocellular cell models.

## 2. Materials and Methods

### 2.1. Materials and Synthesis

1-octadecene, oleylamine, oleic acid, hydrogen tetrachloroaurate (III) hydrate (HAuCl_4_·3H_2_O), iron pentacarbonyl (Fe(CO)_5_), ethanol, toluene and hexane were purchased from Sigma-Aldrich (Darmstadt, Germany). 1,2-distearoyl-sn-glycero-3-phosphoethanolamine-N-[carboxy(polyethylene glycol)-2000] ammonium salt (DSPE-PEG_2000_-COOH) was delivered by Avanti Polar Lipids (Alabaster, AL, USA). Chloroform was purchased from Reachim (Moscow, Russia). All chemicals were of analytical grade.

The Fe_3_O_4_-Au NPs were synthesized following a thermal decomposition method reported elsewhere [1]. A mixture of oleylamine (2 mL; 6 mmol), oleic acid (1.9 mL; 6 mmol) and 1-octadecene (20 mL) were heated to 120 °C under Ar atmosphere. Later, Fe(CO)_5_ (0.3 mL; 2.2 mmol) was injected in the mixture. After 3 min, a tetrachloroaurate (III) hydrate (40 mg; 0.1 mmol) solution in oleylamine (0.5 mL, 1.5 mmol) and 1-octadecene (5 mL) was injected in the mixture. The temperature of the reaction mixture was raised by increments of 3 °C per minute up to 316 °C and there held for 45 min; the mixture was then cooled down to room temperature before exposing it to the air. The precipitate was washed with ethanol, centrifuged and re-dispersed into hexane in the presence of 0.05 mL oleylamine. The suspension was sonicated for 15 min and stored at 4 °C before further use. The amount of surfactant on the surface of NPs was estimated using a thermogravimetric analyzer (TG model 209F3, NETZSCH Holding, Selb, Germany) and found equal to 6% *w/w*.

The Fe_3_O_4_-Au nano-heterostructures were then transferred to aqueous medium by coating with DSPE-PEG_2000_-COOH polymer. To this aim, equal volumes of Fe_3_O_4_-Au NPs and polymer (both 1 mg/mL in chloroform) were mixed in a vial in ultrasonic bath for 5 min and the mixture was left in a weak stream of Ar overnight. After the evaporation of the solvent, 1 mL of deionized (DI) water was added to the precipitate, and the NPs were re-suspended by sonication for 5−10 min; the excess of polymer was removed by double centrifugation for 5 min (RCF 14100g). At the final stage, the NPs were dispersed in 1 mL of DI H_2_O and passed through a syringe filter with a pore diameter of 0.45 μm for sterilization. The NP suspensions are stable upon the storage in 1 × PBS buffer for at least 14 days, during which no visible aggregation or precipitation occurs.

### 2.2. Characterization of NPs

The morphology, average size and size distribution of NPs were determined by a JEOL JEM-1400 transmission electron (TEM) microscope (JEOL Ltd.,Tokyo, Japan) operated at a 120 kV acceleration voltage. Overview images were taken in conventional bright-field TEM mode. The samples were prepared by casting and evaporating a droplet of hexane dispersion onto a carbon-coated copper grid (300 mesh). The average diameter of NPs was evaluated from TEM images by statistical analysis over about 200 NPs for each sample using ImageJ software (NIH, Bethesda, MD, USA) [42]. TEM analysis was performed on the as-prepared samples and after three months to confirm the stability of the nanoparticles. No significant modification in the average size nor in the size distribution of the two components was observed, pointing out the long- term stability of the nano-heterostructures.

The hydrodynamic size of the NPs in water was measured by dynamic light scattering using a Zetasizer Nano ZS (Malvern Panalytical Russia, Moscow, Russia). The average values with error bars were obtained from three measurements of each sample. The hydrodynamic diameter was 107 ± 3 nm, zeta-potential = −13.8 ± 2.0 mV.

The magnetic properties were measured with a SQUID magnetometer (Quantum Design Ltd., San Diego, CA, USA). Hysteresis cycles were recorded at 5 and 300 K in the field range of ±50 kOe. The zero-field cooled and field-cooled (ZFC/FC) magnetizations were obtained by measuring the magnetization as a function of the temperature during heating, after cooling the sample in the presence (M_FC_) and absence (M_ZFC_) of an applied low magnetic field (50 Oe).

Powder X-ray diffraction (XRD) measurements were carried out using a Bruker D8 Advance diffractometer (Bruker, MA, USA) equipped with a Cu Kα radiation (λ = 1.54178 Å) and operating in θ–θ Bragg-Brentano geometry at 40 kV and 40 mA. XRD pattern of the sample was acquired from 25° to 70° (2θ). Lattice parameter and mean crystallite size were evaluated by TOPAS software (Bruker, MA, USA) using the method of a fundamental parameter approach considering a cubic space group Fd3$\bar{3}$m.

The optical properties of nano-heterostructures were evaluated by UV-Vis spectroscopy, using a Jasco V-670 double-beam spectrophotometer (JASCO Deutschland GmbH, Pfungstadt, Germany) covering a wavelength range from 300 to 1300 nm.

To evaluate the hyperthermic efficiency of Fe_3_O_4_-Au NPs, calorimetric measurement of Specific Absorption Rate (SAR) was performed using a 6 kW power supply by Fives Celes^®^ (Lautenbach, France). Measurements were carried out by applying an alternating magnetic field of 17 kA/m amplitude (H_0_) and 183 kHz frequency (f) on a dispersion in toluene of Fe_3_O_4_-Au NPs (8.0 mg/mL). The H_0_ and f values, were chosen such as their product is below the tolerance limit (H_0_ × f = 5 × 10^9^ Am^−1^·s^−1^), currently accepted to avoid any undesired side effects on human beings for small region exposure [43]. The temperature of the sample was recorded using an optical fiber temperature probe (OPTOCON, Dresden, Germany). Samples were surrounded by polystyrene and hosted in a glass Dewar to thermally isolate the sample from the surroundings.

### 2.3. Cytotoxicity, Relaxivity and Biological Assessment

Human hepatocellular carcinoma cell lines Huh7 were obtained from the Japanese Collection of Research Bioresources (JCRB, Osaka, Japan), Alexander (PLC/PRF/5, American Type Culture Collection- ATCC, Manassas, VA, USA) and cultured in Eagle’s Minimum Essential Medium (EMEM) (ATCC, Manassas, VA, USA) supplemented with 10% fetal bovine serum (FBS, Thermo Fisher Scientific, Waltman, MA, USA) as recommended by the supplier. The cultures were incubated in a humidified 5% CO_2_ atmosphere at 37 °C.

HepG2 hepatoblastoma-derived cells were maintained in Dulbecco’s modified Eagle’s medium: Nutrient Mixture F-12 (DMEM/F-12) medium (Gibco, Waltham, MA, USA) supplemented with 10% Fetal Bovine Serum (Sigma, St. Louis, MO, USA), 2 mM L-glutamine (Gibco, Waltham, MA, USA), antibiotics (0.1 U/mL penicillin and 0.1 μg/mL streptomycin; Gibco, Waltham, MA, USA). The cells were cultured at 37 °C in a humidified incubator supplied with 5% CO_2_.

The cytotoxicity of Fe_3_O_4_-Au NPs was tested in human hepatic cell lines (Huh7 and PLC/PRF/5-Alexander) at different concentrations: 0.1, 10 and 100 μg/mL. Cytotoxicity was assessed by WST-1 assay (Sigma, St. Louis, MO, USA). The detection time points were 1, 2, 4, 6, 8, 14, 24, 48 and 72 h. Cells cultivated in medium without nanoparticles were used as a control.

T_2_ relaxation rate of water protons in the presence of Fe_3_O_4_-Au NPs and in HepG2 cells was measured in 500 μL test tubes at 18 °C in a ClinScan 7 T MRI system (Bruker BioSpin, MA, USA). Image acquisition was performed in the Spin Echo mode with following parameters: MRI system TR = 10 s, TE = 16, 24, …, 256 ms, flip angle = 180°, base resolution 448 × 640 pixel, field of view 84 × 120 mm^2^. Signal intensities from regions of interest were determined using ImageJ, and the T_2_ relaxation time was calculated by exponential fitting as a function of TE. The r_2_ relaxivity values were calculated from the linear fitting of T_2_^−1^ relaxation times as a function of Fe concentration, evaluated by in water and in HepG2 cells. The slopes represent the r_2_ values for Fe_3_O_4_-Au NPs in water and in HepG2 cell culture used for MR imaging. For the latter experiment, cells were incubated with NPs (100 μg·mL^−1^ Fe_3_O_4_) during 24 h and non-bound NPs were removed by cell washing with PBS. Cells with attached NPs were suspended in 2% agarose gel.

Total proteins were obtained from whole cells lysate using Radioimmunoprecipitation assay buffer (RIPA) by standard manufactured protocol (Thermoscientific, Waltham, MA, USA). Protein samples were subjected to Sodium Dodecyl Sulphate - PolyAcrylamide Gel Electrophoresis (SDS-PAGE), transferred to Polyvinylidene difluoride (PVDF) membranes. The membranes were blocked with 5% (*w/v*) nonfat dried milk for 1 h, then incubated with specific primary antibody of Caspase 3 (diluted 1:1000 in 5% (*w/v*) nonfat dried milk) (Cell Signaling, Leiden, The Netherlands) overnight. The secondary antibody was diluted according to manufacture recommendation in the appropriate blocking buffer. Finally, to visualize the protein of interest, membrane was covered with enhanced chemiluminescence (ECL) luminol reagent (GE Healthcare, Chicago, IL, USA). The light emission was detected by a luminescent image analyzer LAS-4000 Mini detection system (GE Healthcare, Uppsala, Sweden) or ImageQuant LAS4000 (GE Healthcare, Uppsala, Sweden).

## 3. Results and Discussions

### 3.1. Physical Properties of the Nano-Heterostructures

**Figure 1:**
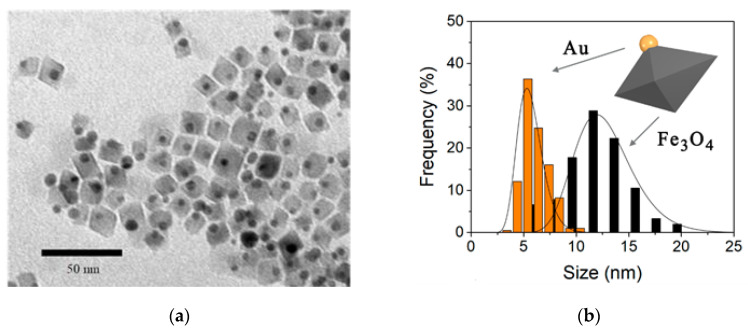
(**a**) Transmission electron microscopy (TEM) image of Fe_3_O_4_-Au and (**b**) size distribution of the gold (red bars, sphere diameter) and magnetite (blue bars, octahedra diagonal) components.

The structure and morphology of the Fe_3_O_4_-Au NPs were checked by transmission electron microscopy and X-ray diffraction. Figure 1 presents a TEM image representative of the sample; it consists almost entirely of dimeric NPs made up of a gold sphere and a well-defined magnetite octahedron, combined in the 1:1 ratio. The average size and standard deviation were estimated by fitting the experimental histograms to the log-normal function [44]. The Fe_3_O_4_ NPs median diagonal length is 12 ± 4 nm and the average diameter of the spherical Au NPs is 6 ± 1 nm.

It deserves to be mentioned that the formation of regularly shaped magnetite octahedra with average size as those here observed is rather uncommon. Below 15−20 nm, indeed, the dominant role played by surface energy favors the growth of spherically shaped nanograins, which exhibit the smallest surface area [45,46,47]. The formation of small octahedrons of Fe_3_O_4_ seems to be favored by the presence of pristine nuclei of noble metal (which in our case form in the very first step of the one-pot reaction) and by long reaction time and high temperature [48,49].

**Figure 2:**
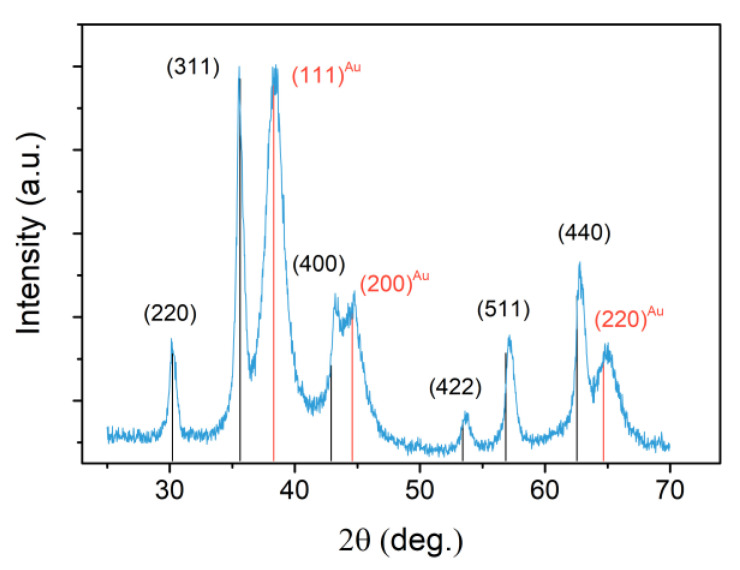
X-Ray Diffraction (XRD) pattern of Fe_3_O_4_-Au nanoparticles (NPs) (blue line) and reference patterns of magnetite (black bars; JCPDS 04-007-2718) and gold (red bars; JCPDS 00-004-0784).

Figure 2 shows the XRD pattern for the heterodimeric NPs, where the peak typical of a spinel ferrite and of Au can be identified, (peaks at 2θ = 30.19°, 35.56°, 43.29°, 53.69°, 57.16° and 62.82° for magnetite and at 2θ = 38.40°, 44.59°, 64.95° for gold). No impurity peaks corresponding to other iron oxide phases such as wustite, hematite or FeOOH can be recognized in the pattern. XRD data analysis revealed that the lattice parameter was a = 4.066(1) for the metal, and a = 8.387(1) for the oxide phase. This latter value was much closer to that expected for magnetite than for the fully oxidezed counterpart, maghemite, suggesting no relevant oxidation occurred. The average crystallite size was found equal to 5 ± 1 nm and 14 ± 1 nm for Au and Fe_3_O_4_, respectively, in good agreement with TEM data, revealing the single-crystal nature and high crystallinity of both components.

**Figure 3:**
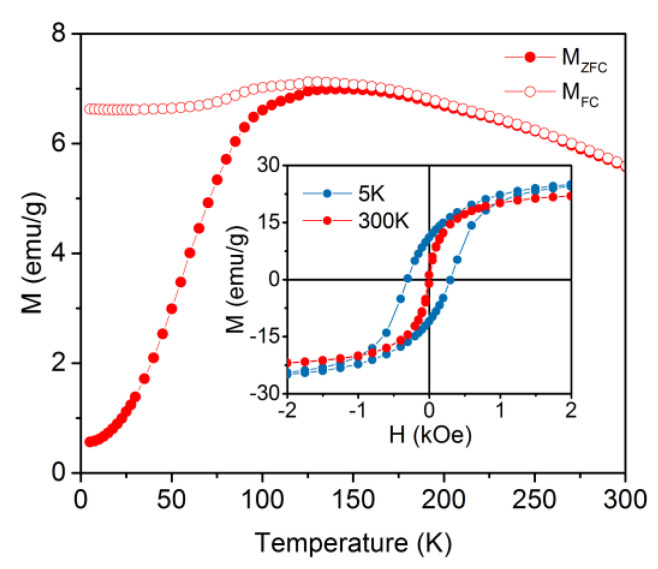
Zero-field cooled and field-cooled (ZFC/FC) magnetizations of Fe_3_O_4_-Au NPs. In the inset, the hysteresis loops at 5 and 300 K in the low magnetic field range are reported.

The hysteresis loop measured at 300 K (inset Figure 3) suggests a superparamagnetic behavior with no magnetic irreversibility and a saturation magnetization M_S_ = 24 ± 2 emu/g. At 5 K, the sample is in a “blocked regime” characterized by a considerable value of the coercive field (300 Oe) and M_S_ = 31 ± 3 emu/g. The low M_S_ value is due to the diamagnetic contribution of gold and of the organic shell. Indeed, when rescaled for the weight percentage of magnetite in the NPs, M_S_ becomes 82 ± 5 emu/g, which is in the range expected for crystalline magnetite (80−92 emu/g [50]).

The reversibility of the ZFC/FC magnetization curves above 140 K confirms the superparamagnetic nature of the particles at room temperature. The irreversibility temperature (T_irr_), determined as temperature when M_FC_ and M_ZFC_ curves merge [51,52], was found at about 140 K. Above T_irr_, all the particles are in the superparamagnetic state characterized by the presence of the thermal reversibility in the magnetic behavior (absence of remanent magnetization). With the decreasing of temperature below T_irr_, the particles are becoming “blocked” and present a ferro (i) magnetic-like behavior. A small bump was observed at approximately 125 K, which corresponds to the Verwey transition from monoclinic to the cubic inverse spinel structure of magnetite [53], further confirming that magnetite has not considerably oxidized. The low-temperature plateau in the FC curve can be attributed to the strong dipolar interactions among particles occurring when the sample is in the powder state [54].

**Figure 4:**
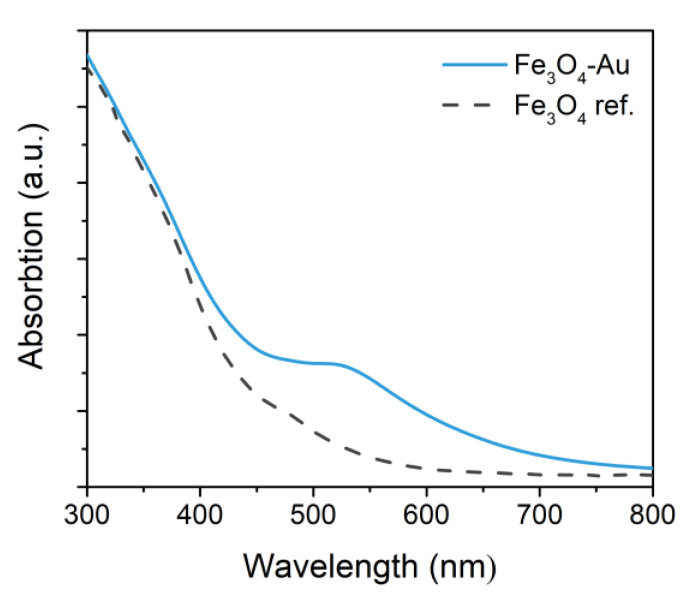
Extinction spectrum of Fe_3_O_4_-Au NPs measured on a toluene suspension and reference spectrum for the bare Fe_3_O_4_ NPs from [57].

Figure 4 shows the UV-vis spectrum of the Fe_3_O_4_-Au particles dispersed in toluene acquired from 300 to 800 nm. The localized surface plasmon resonance peak, characteristic of gold nanoparticles, can be recognized at 520 ± 10 nm. The peak, which is partially dumped due to the proximity of the magnetic counterpart, is not red shifted with respect to what is commonly observed for Au nanocrystals with a size below 20 nm [55], suggesting the two components share a small interface [55,56].

**Figure 5:**
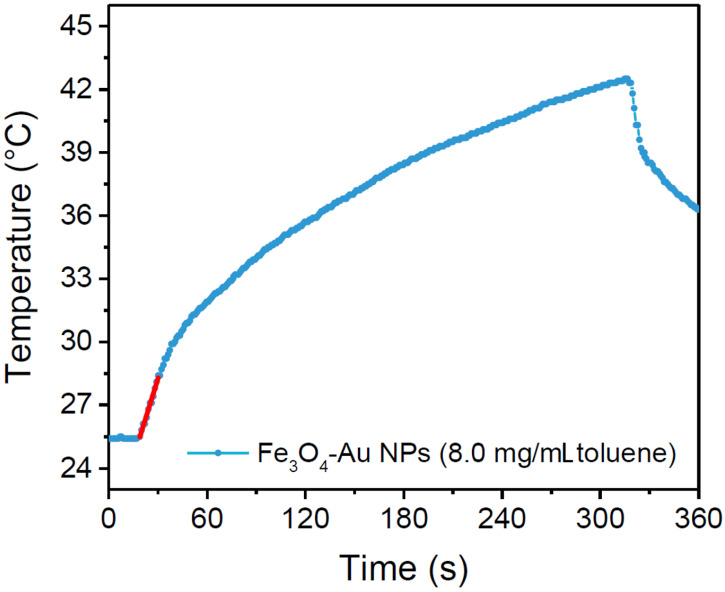
Temperature kinetics of a Fe_3_O_4_-Au NPs suspension in toluene (8.0 mg/mL) acquired applying for 300 s an alternating magnetic field of 17 kA/m amplitude (H_0_) and 183 kHz frequency (f).

The hyperthermic efficiency of the Fe_3_O_4_-Au NPs dispersion in toluene was tested. To quantify the heating capability, the SAR value was evaluated using the following equation:

(1) $$SAR=\frac{\sum_{i}m_{i}c_{pi}}{m_{Me}}\frac{\Delta T}{\Delta t},$$

where Δ*T* is the temperature increase in the interval of time Δ*t*, *m_Me_* is the total mass of metal, *m_i_* is the mass of the i-species and *C_pi_* its specific heat. The sum is extended to all the i-species involved in the heat exchange. Since the measurement was carried in non-adiabatic conditions, the Δ*T/*Δ*t* value was extrapolated for t → 0 by considering the initial slope of the temperature kinetic curves. From the experimental curve (Figure 5) a rapid temperature increase of ca. 17 °C in 300 s was observed and the corresponding SAR was estimated 227 W/g_Fe_. The obtained SAR value is remarkably high, considering the small size of the iron oxide part [58,59]. This result is likely due to the high crystalline quality of the material, which leads to the most stable octahedral geometry. The observed behavior suggests our nano-heterostructures are suitable candidates to be used as heat mediators for hyperthermia treatment.

### 3.2. Cytotoxicity Tests

**Figure 6:**
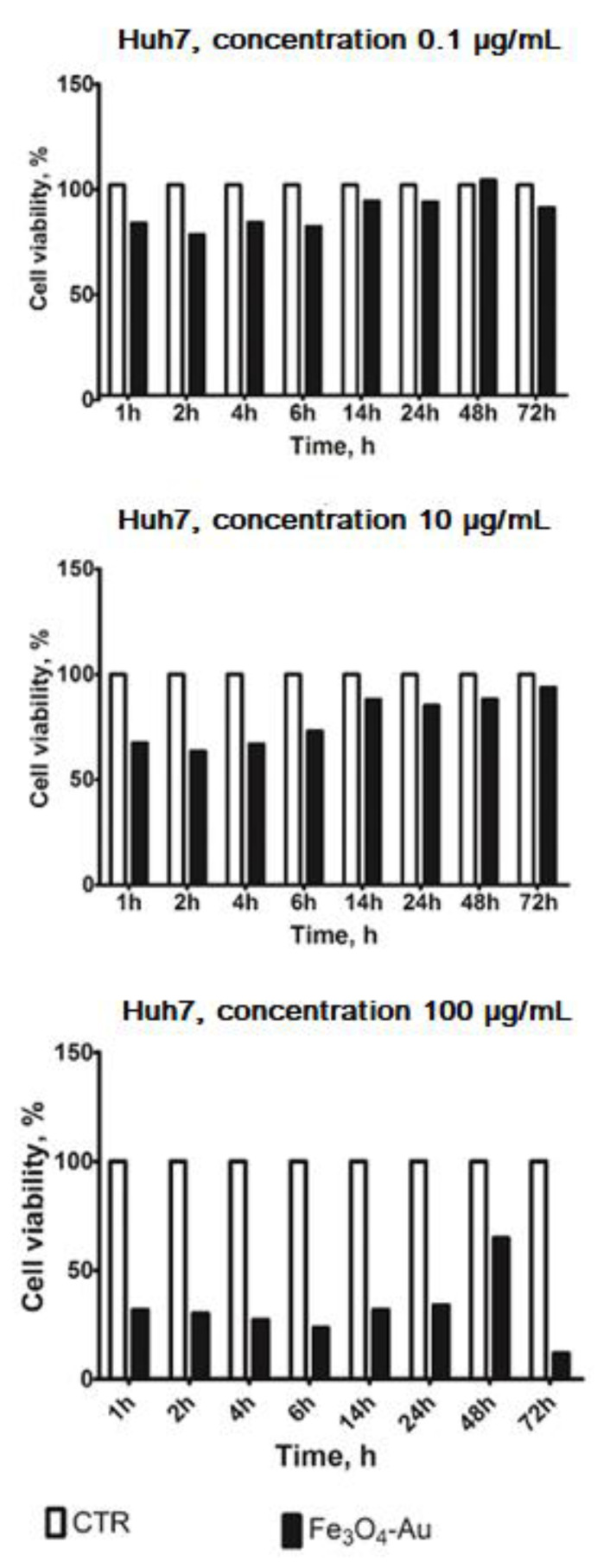
Huh7 cell viability after Fe_3_O_4_-Au NP incubation at increasing time steps and different concentrations.

Since inorganic NPs predominantly accumulate in the liver after i.v. administration, this organ was chosen as the target to investigate the contrast enhancement capability of our nanoheterostructures. In the first step, the human HCC Huh7 cell line was used as a model for the assessment of NP-induced liver toxicity. These cells were selected since they are the most sensitive to cytotoxicity among liver cells [60]. Cell death kinetics upon NP treatment is shown in Figure 6. A pilot experiment (single run) was performed to optimize the concentration of nanoparticles and the exposure time. A significant reduction in the cell viability was observed at 100 μg/mL concentration, while lower concentrations induced only a slight decrease.

**Figure 7:**
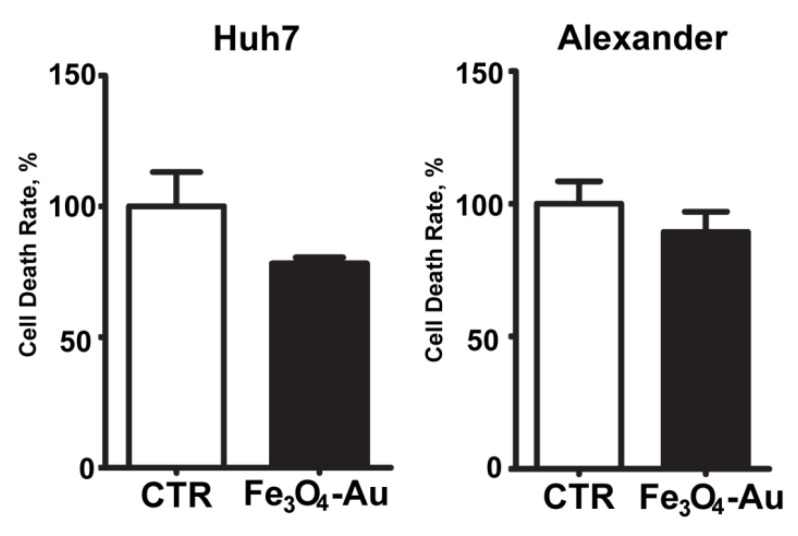
Cell line variability: comparison of two different hepatoma cell lines after a 6 h injection of Fe_3_O_4_-Au NPs at 10 ug/mL. The data are expressed as means ± SEM (n = 3).

To validate cytotoxicity results, we utilized another cell line (PLC/PRF/5-Alexander). It is noticeable that no significant difference between control cells and cells incubated with nanoparticles (10 μg/mL, 6 h) appeared in the Alexander cell line (Figure 7), confirming the results obtained for Huh7.

**Figure 8:**
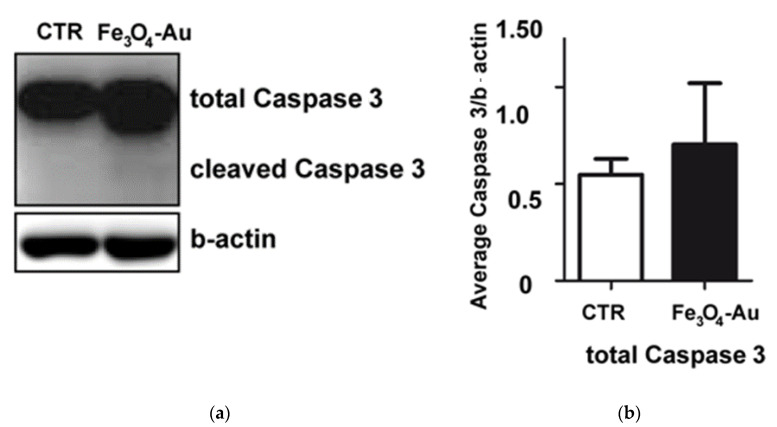
Western immunoblot analysis of the total and cleaved form of Caspase 3 in the Huh7 cell line. (**a**) Immunoblotting caspase 3 and b-actin. (**b**) Densitometric quantification of the total form of Caspase 3. Average band intensity after Western blotting of Caspase 3/b-actin. The data are expressed as means ± SEM (n = 3).

For a more detailed analysis, we verified the effect of Fe_3_O_4_-Au nanoparticles on cell death activation by the immunoblotting of apoptosis marker—the cleaved form of Caspase 3 (Figure 8).

After a 6 h incubation, Fe_3_O_4_-Au NPs did not activate the cleavage of Caspase 3 (dilution 1:1000) in the Huh7 cell line. Quantification of the immunobloting demonstrated no significant difference in the total form of Caspase 3 between control samples and samples incubated with nanoparticles. The cleaved form of Caspase 3 was not detectable. Accordingly, the Fe_3_O_4_-Au nanoparticles showed low cytotoxicity effects on HepG2 and Alexander cells’ culture, too, confirming their potential use in biomedical application as diagnostic tool for liver diseases.

### 3.3. MRI Experiments

**Figure 9:**
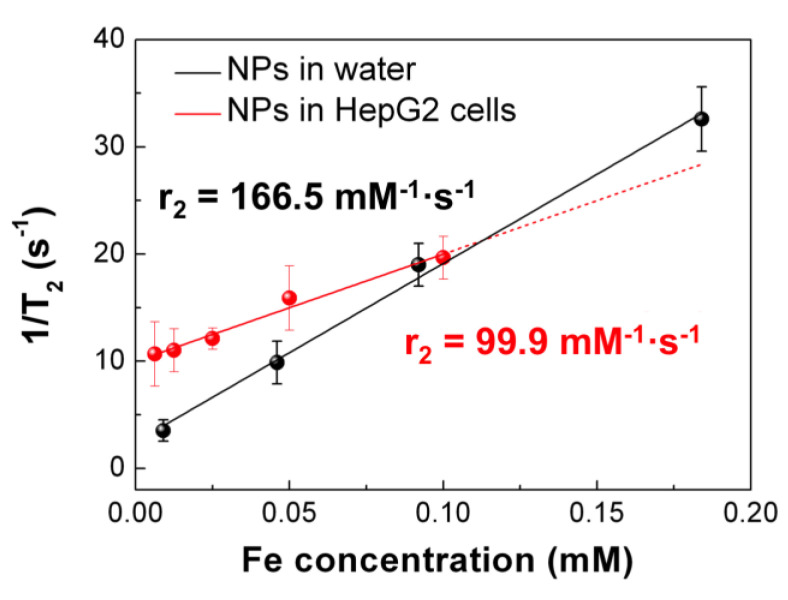
Proton T_2_ relaxation time in magnetic resonance imaging (MRI) as a function of iron concentration for NPs in water and in HepG2 cells. The r_2_ value is determined by the slope of the linear fit.

Finally, we investigated the capability of Fe_3_O_4_-Au NPs to act as a MRI T_2_-contrast agent, by measuring r_2_ relaxivity values in vitro in water solutions and HepG2 cells. MR imaging of phantoms composed of serial dilutions of Fe_3_O_4_-Au NPs demonstrated the linearity of T_2_-weighted MR signal with increasing iron concentrations (Figure 9). The transverse relaxivity coefficient r_2_ determined by the slope of the linear fitting curve was 166.5 mM^−1^·s^−1^ for Fe_3_O_4_-Au NPs in water solution. This value is slightly higher than commercial T_2_ contrast agents (r_2_ ≈ 160 mM^−1^·s^−1^) [61]. Interestingly, the transverse relaxivity measured in water nicely agrees with the general trend observed by Efremova et al. for a series of Fe_3_O_4_-Au NPs, with similar morphology, and different average size of the magnetic component [39].

After the incubation of HepG2 cells with the NPs for 24 h, we observed a 40% decrease in r_2_ (99.9 mM^−1^·s^−1^), which is in line with other reports on the MRI properties of Fe_3_O_4_-Au NPs in cell cultures [6,62]. For example, in [62], the r_2_ value decreased from 105 mM^−1^·s^−1^ for the suspension of NPs to 80 mM^−1^·s^−1^ when the latter were incubated with A431 cells, as the proton relaxivity decreases upon the cellular compartmentalization of the NPs. Together with a possible aggregation, partial oxidation or dissolution of the NPs can also be the reason for the r_2_ decrease. Concerning the transverse relaxivity value, in a recent study by Maniglio et al. [63], r_2_ = 147 mM^−1^·s^−1^ for Fe_3_O_4_-Au flower-like NPs in water solution was reported. In that work, in vitro studies were also carried out on MG63 and 3T3 cell cultures, demonstrating the synergistic effect of the Fe_3_O_4_-Au flower-like NPs and x-ray radiation in MG63 cell culture. Therefore, the authors suggest these NPs as a theranostic tool for MRI-guided radiosensitization.

Intriguing in vivo studies reported the use of composite Fe_3_O_4_-Au NPs (core-cluster) for liver diagnostics by MRI and CT [64], showing r_2_ =146 mM^−1^·s^−1^. This value is consistent with our data, but a dumbbell-like structure like that of our NPs offers the advantage to allow a more precise control over Fe/Au mass ratio and the relative surface area of both components. Analogously, Zhao et al. [65] investigated Fe_3_O_4_-Au strawberry-like NPs with the r_2_ value of 80 mM^−1^·s^−1^ in water solution and demonstrated the effectiveness of such hybrids in vivo for multimodal (MRI and CT) imaging of various liver diseases. Other works reported the MRI performance of core-shell Fe_3_O_4_-Au NPs in vivo in tumor-bearing animals, but not in the liver, and leaving the question of biodegradability of core-shell NPs open [66,67].

Finally, it should be noted that most of the studies reported so far are conducted at the clinically relevant magnetic fields (3 T), but sometimes higher fields (4−7 T) are used, like in our case. Indeed, Smolensky et al. [68] have shown that the r_2_ of iron oxide NPs is independent of the magnetic field strength in the frequency range of 20–500 MHz. The results of relaxivity measurement obtained at 7 T (298.06 MHz), which has a high signal-to-noise ratio and a high spatial resolution, are thus also valid for clinical scanners at lower fields.

In this framework, the Fe_3_O_4_-Au hybrids obtained in this study have higher r_2_ values than other heterodimeric or core-shell Fe_3_O_4_-Au NPs currently considered for the imaging of liver cells in vitro and thus have a high potential for further in vivo investigations. The observed high efficacy can be ascribed to the high crystallinity (and thus good magnetic properties) and octahedral shape of our hybrid NPs.

## 4. Conclusions

In this work, Fe_3_O_4_-Au nanoheterostructures with an average size of 12 ± 4 nm and 6 ± 1 nm for magnetite and gold counterparts, respectively, were synthesized. The obtained NPs showed superparamagnetic behavior with blocking temperature of about 140 K and thus no residual magnetization at room temperature. The saturation magnetization value (82 ± 5 emu/g), close to the bulk value of magnetite, and the presence of the Verwey transition at about 125 K, demonstrated the high purity of the magnetic phase. The high crystallinity and average size of the magnetic component confer the Fe_3_O_4_-Au NPs the proper magnetic properties to be excellent heat mediators for magnetic fluid hyperthermia, with an SAR value of 227 W/g_Fe_. The NPs were functionalized with DSPE-PEG_2000_-COOH and showed a low level of liver cancer cell cytotoxicity. Cell viability was reduced only at the highest concentration tested (100 μg/mL). The possibility to use Fe_3_O_4_-Au NPs as MRI T_2_-contrast agents for liver was demonstrated in vitro in a water solution containing HepG2 cells, even though a 40% reduction in r_2_ value was observed in cell culture, as already reported in previous works. This study indicates that this type of hybrid NP is a good candidate for theranostics of liver cancer, as it can be used as an MRI-contrast agent and, potentially, as a heat mediator for magnetic fluid hyperthermia. On the other hand, the presence of the plasmonic components makes this hybrid nanostructures appealing also for different applications such as magnetically assisted optical sensing. Moreover, the gold surface can be further functionalized with other biomolecules to carry additional functionality.

Due to the high relaxometric efficiency and the non-significant hepatotoxicity of the heterodimeric nanoparticles, additional in vitro tests will be carried out in the near future to assess their efficacy also as contrast agents for CT and as magnetically assisted optical sensors, as well as to evaluate their antitumor activity for liver cancer treatment.

## References

[1] Yu H., Chen M., Rice P.M., Wang S.X., White R.L., Sun S. (2005). Dumbbell-like bifunctional Au-Fe_3_O_4_ nanoparticles. Nano Lett..

[2] Frey N.A., Phan M.H., Srikanth H., Srinath S., Wang C., Sun S. (2009). Interparticle interactions in coupled Au-Fe_3_O_4_ nanoparticles. J. Appl. Phys..

[3] Leung K.C.F., Xuan S., Zhu X., Wang D., Chak C.P., Lee S.F., Ho W.K.W., Chung B.C.T. (2012). Gold and iron oxide hybrid nanocomposite materials. Chem. Soc. Rev..

[4] Liu S., Guo S., Sun S., You X.Z. (2015). Dumbbell-like Au-Fe_3_O_4_ nanoparticles: A new nanostructure for supercapacitors. Nanoscale.

[5] Fantechi E., Roca A.G., Sepúlveda B., Torruella P., Estradé S., Peiró F., Coy E., Jurga S., Bastús N.G., Nogués J. (2017). Seeded Growth Synthesis of Au-Fe_3_O_4_ Heterostructured Nanocrystals: Rational Design and Mechanistic Insights. Chem. Mater..

[6] Jiang W., Huang Y., An Y., Kim B.Y.S. (2015). Remodeling Tumor Vasculature to Enhance Delivery of Intermediate-Sized Nanoparticles. ACS Nano.

[7] Sotiriou G.A., Starsich F., Dasargyri A., Wurnig M.C., Krumeich F., Boss A., Leroux J.C., Pratsinis S.E. (2014). Photothermal killing of cancer cells by the controlled plasmonic coupling of silica-coated Au/Fe_2_O_3_ nanoaggregates. Adv. Funct. Mater..

[8] Espinosa A., Bugnet M., Radtke G., Neveu S., Botton G.A., Wilhelm C., Abou-Hassan A. (2015). Can magneto-plasmonic nanohybrids efficiently combine photothermia with magnetic hyperthermia?. Nanoscale.

[9] Bao J., Chen W., Liu T., Zhu Y., Jin P., Wang L., Liu J., Wei Y., Li Y. (2007). Bifunctional Au-Fe_3_O_4_ nanoparticles for protein separation. ACS Nano.

[10] Xu C., Wang B., Sun S. (2009). Dumbbell-like Au-Fe_3_O_4_ Nanoparticles for Target-Specific Platin Delivery. J. Am. Chem. Soc..

[11] Zhang X., Dong S. (2018). Synthesis and Application of Au-Fe_3_O_4_ Dumbbell-Like Nanoparticles.

[12] Lee J.H., Huh Y.M., Jun Y.W., Seo J.W., Jang J.T., Song H.T., Kim S., Cho E.J., Yoon H.G., Suh J.S. (2007). Artificially engineered magnetic nanoparticles for ultra-sensitive molecular imaging. Nat. Med..

[13] McDonagh B.H., Singh G., Hak S., Bandyopadhyay S., Augestad I.L., Peddis D., Sandvig I., Sandvig A., Glomm W.R. (2016). L -DOPA-Coated Manganese Oxide Nanoparticles as Dual MRI Contrast Agents and Drug-Delivery Vehicles. Small.

[14] Haun J.B., Yoon T.J., Lee H., Weissleder R. (2010). Magnetic nanoparticle biosensors. Wiley Interdiscip. Rev. Nanomed. Nanobiotechnol..

[15] Pariti A., Desai P., Maddirala S.K.Y., Ercal N., Katti K.V., Liang X., Nath M. (2014). Superparamagnetic Au-Fe_3_O_4_ nanoparticles: One-pot synthesis, biofunctionalization and toxicity evaluation. Mater. Res. Express.

[16] Xie H., Zhu Y., Jiang W., Zhou Q., Yang H., Gu N., Zhang Y., Xu H., Xu H., Yang X. (2011). Lactoferrin-conjugated superparamagnetic iron oxide nanoparticles as a specific MRI contrast agent for detection of brain glioma in vivo. Biomaterials.

[17] Panchapakesan B., Wickstrom E. (2007). Nanotechnology for Sensing, Imaging, and Treating Cancer. Surg. Oncol. Clin. N. Am..

[18] Socoliuc V., Peddis D., Petrenko V.I., Avdeev M.V., Susan-resiga D., Szabó T., Turcu R., Tombácz E., Vékás L. (2019). Magnetic Nanoparticle Systems for Nanomedicine—A Materials Science Perspective. Magetochemistry.

[19] Stoeva S.I., Huo F., Lee J.S., Mirkin C.A. (2005). Three-layer composite magnetic nanoparticle probes for DNA. J. Am. Chem. Soc..

[20] Laurent S., Dutz S., Häfeli U.O., Mahmoudi M. (2011). Magnetic fluid hyperthermia: Focus on superparamagnetic iron oxide nanoparticles. Adv. Colloid Interface Sci..

[21] Guisasola E., Asín L., Beola L., de la Fuente J.M., Baeza A., Vallet-Regí M. (2018). Beyond traditional hyperthermia: In vivo cancer treatment with magnetic-responsive mesoporous silica nanocarriers. ACS Appl. Mater. Interfaces.

[22] Williams H.M. (2017). The application of magnetic nanoparticles in the treatment and monitoring of cancer and infectious diseases. Biosci. Horiz. Int. J. Stud. Res..

[23] Blanco-Andujar C., Teran F.J., Ortega D. (2018). Current Outlook and Perspectives on Nanoparticle-Mediated Magnetic Hyperthermia. Iron Oxide Nanoparticles for Biomedical Applications.

[24] Martinkova P., Brtnicky M., Kynicky J., Pohanka M. (2018). Iron Oxide Nanoparticles: Innovative Tool in Cancer Diagnosis and Therapy. Adv. Healthc. Mater..

[25] Panchapakesan B., Book-Newell B., Sethu P., Rao M., Irudayaraj J. (2011). Gold nanoprobes for theranostics. Nanomedicine.

[26] Kumar D., Soni R.K., Ghai D.P. (2019). Pulsed photoacoustic and photothermal response of gold nanoparticles. Nanotechnology.

[27] Xi D., Dong S., Meng X., Lu Q., Meng L., Ye J. (2012). Gold nanoparticles as computerized tomography (CT) contrast agents. Rsc. Adv..

[28] Mahan M.M., Doiron A.L. (2018). Gold Nanoparticles as X-Ray, CT, and Multimodal Imaging Contrast Agents: Formulation, Targeting, and Methodology. J. Nanomater..

[29] Hamdy M.E., Del Carlo M., Hussein H.A., Salah T.A., El-Deeb A.H., Emara M.M., Pezzoni G., Compagnone D. (2018). Development of gold nanoparticles biosensor for ultrasensitive diagnosis of foot and mouth disease virus. J. Nanobiotechnol..

[30] Zhu Z. (2016). Gold nanoparticle based biosensors. New Dev. Gold Nanomater. Res..

[31] Thompson D.T. (2007). Using gold nanoparticles for catalysis. Nano Today.

[32] Grisel R., Weststrate K.J., Gluhoi A., Nieuwenhuys B.E. (2002). Catalysis by gold nanoparticles. Gold Bull..

[33] Nguyen T.T., Mammeri F., Ammar S. (2018). Iron oxide and gold based magneto-plasmonic nanostructures for medical applications: A review. Nanomaterials.

[34] Tran V.T., Kim J., Tufa L.T., Oh S., Kwon J., Lee J. (2018). Magnetoplasmonic Nanomaterials for Biosensing/Imaging and in Vitro/in Vivo Biousability. Anal. Chem..

[35] Moghadam F.F. (2017). Using nanoparticles in medicine for liver cancer imaging. Oman Med. J..

[36] Taghizadeh S., Alimardani V., Roudbali P.L., Ghasemi Y., Kaviani E. (2019). Gold nanoparticles application in liver cancer. Photodiagnosis Photodyn. Ther..

[37] Wilhelm S., Tavares A.J., Dai Q., Ohta S., Audet J., Dvorak H.F., Chan W.C.W. (2016). Analysis of nanoparticle delivery to tumours. Nat. Rev. Mater..

[38] Efremova M.V., Naumenko V.A., Spasova M., Garanina A.S., Abakumov M.A., Blokhina A.D., Melnikov P.A., Prelovskaya A.O., Heidelmann M., Li Z.A. (2018). Magnetite-Gold nanohybrids as ideal all-in-one platforms for theranostics. Sci. Rep..

[39] Efremova M.V., Nalench Y.A., Myrovali E., Garanina A.S., Grebennikov I.S., Gifer P.K., Abakumov M.A., Spasova M., Angelakeris M., Savchenko A.G. (2018). Size-selected Fe_3_O_4_-Au hybrid nanoparticles for improved magnetism-based theranostics. Beilstein J. Nanotechnol..

[40] Levada K., Pshenichnikov S., Omelyanchik A., Rodionova V., Nikitin A., Savchenko A., Schetinin I., Zhukov D., Abakumov M., Majouga A. (2020). Progressive lysosomal membrane permeabilization induced by iron oxide nanoparticles drives hepatic cell autophagy and apoptosis. Nano Converg..

[41] Patil R.M., Thorat N.D., Shete P.B., Bedge P.A., Gavde S., Joshi M.G., Tofail S.A.M., Bohara R.A. (2018). Comprehensive cytotoxicity studies of superparamagnetic iron oxide nanoparticles. Biochem. Biophys. Rep..

[42] Schneider C.A., Rasband W.S., Eliceiri K.W. (2012). NIH Image to ImageJ: 25 years of image analysis. Nat. Methods.

[43] Hergt R., Dutz S. (2007). Magnetic particle hyperthermia-biophysical limitations of a visionary tumour therapy. J. Magn. Magn. Mater..

[44] Muscas G., Jovanović S., Vukomanović M., Spreitzer M., Peddis D. (2019). Zn-doped cobalt ferrite: Tuning the interactions by chemical composition. J. Alloys Compd..

[45] Muro-Cruces J., Roca A.G., López-Ortega A., Fantechi E., Del-Pozo-Bueno D., Estradé S., Peiró F., Sepúlveda B., Pineider F., Sangregorio C. (2019). Precise Size Control of the Growth of Fe_3_O_4_ Nanocubes over a Wide Size Range Using a Rationally Designed One-Pot Synthesis. ACS Nano.

[46] López-Ortega A., Lottini E., Fernández C.D.J., Sangregorio C. (2015). Exploring the Magnetic Properties of Cobalt-Ferrite Nanoparticles for the Development of a Rare-Earth-Free Permanent Magnet. Chem. Mater..

[47] Chen C.-J., Chiang R.-K., Wang J.-S., Wang S.-L. (2013). Synthesis and magnetic properties of octahedral magnetite nanoparticles in 20–110 nm range. J. Nanopart. Res..

[48] Han C.W., Choksi T., Milligan C., Majumdar P., Manto M., Cui Y., Sang X., Unocic R.R., Zemlyanov D., Wang C. (2017). A Discovery of Strong Metal-Support Bonding in Nanoengineered Au-Fe_3_O_4_ Dumbbell-like Nanoparticles by in Situ Transmission Electron Microscopy. Nano Lett..

[49] Nalench Y.A., Shchetinin I.V., Skorikov A.S., Mogilnikov P.S., Farle M., Savchenko A.G., Majouga A.G., Abakumov M.A., Wiedwald U. (2020). Unravelling the nucleation, growth, and faceting of magnetite-gold nanohybrids. J. Mater. Chem. B.

[50] Schieber M.M. (1967). Experimental Magnetochemistry: Nonmetallic Magnetic Materials.

[51] Omelyanchik A., Salvador M., D’Orazio F., Mameli V., Cannas C., Fiorani D., Musinu A., Rivas M., Rodionova V., Varvaro G. (2020). Magnetocrystalline and Surface Anisotropy in CoFe_2_O_4_ Nanoparticles. Nanomaterials.

[52] Muscas G., Peddis D., Cobianchi M., Lascialfari A., Cannas C., Musinu A., Omelyanchik A., Rodionova V., Fiorani D., Mameli V. (2019). Magnetic Interactions Versus Magnetic Anisotropy in Spinel Ferrite Nanoparticles. IEEE Magn. Lett..

[53] Walz F. (2002). The Verwey transition—A topical review. J. Phys. Condens. Matter.

[54] Peddis D., Cannas C., Musinu A., Ardu A., Orrù F., Fiorani D., Laureti S., Rinaldi D., Muscas G., Concas G. (2013). Beyond the Effect of Particle Size: Influence of CoFe_2_O_4_ Nanoparticle Arrangements on Magnetic Properties. Chem. Mater..

[55] Bohren C.F., Huffman D.R. (1998). Absorption and Scattering of Light by Small Particles.

[56] Omelyanchik A., Efremova M., Myslitskaya N., Zybin A., Carey B.J., Sickel J., Kohl H., Bratschitsch R., Abakumov M., Majouga A. (2019). Magnetic and Optical Properties of Gold-Coated Iron Oxide Nanoparticles. J. Nanosci. Nanotechnol..

[57] Fantechi E., Innocenti C., Bertoni G., Sangregorio C., Pineider F. (2020). Modulation of the magnetic properties of gold-spinel ferrite heterostructured nanocrystals. Nano Res..

[58] Lartigue L., Innocenti C., Kalaivani T., Awwad A., Duque M.D.M.S., Guari Y., Larionova J., Gueírin C., Montero J.L.G., Barragan-Montero V. (2011). Water-dispersible sugar-coated iron oxide nanoparticles. An evaluation of their relaxometric and magnetic hyperthermia properties. J. Am. Chem. Soc..

[59] Guardia P., Di Corato R., Lartigue L., Wilhelm C., Espinosa A., Garcia-Hernandez M., Gazeau F., Manna L., Pellegrino T. (2012). Water-soluble iron oxide nanocubes with high values of specific absorption rate for cancer cell hyperthermia treatment. ACS Nano.

[60] Smolková B., Lunova M., Lynnyk A., Uzhytchak M., Churpita O., Jirsa M., Kubinová Š., Lunov O., Dejneka A. (2019). Non-thermal plasma, as a new physicochemical source, to induce redox imbalance and subsequent cell death in liver cancer cell lines. Cell. Physiol. Biochem..

[61] Basini M., Guerrini A., Cobianchi M., Orsini F., Bettega D., Avolio M., Innocenti C., Sangregorio C., Lascialfari A., Arosio P. (2019). Tailoring the magnetic core of organic-coated iron oxides nanoparticles to influence their contrast efficiency for Magnetic Resonance Imaging. J. Alloys Compd..

[62] Xu C., Xie J., Ho D., Wang C., Kohler N., Walsh E.G., Morgan J.R., Chin Y.E., Sun S. (2008). Au-Fe_3_O_4_ dumbbell nanoparticles as dual-functional. Angew. Chem. Int. Ed..

[63] Maniglio D., Benetti F., Minati L., Jovicich J., Valentini A., Speranza G., Migliaresi C. (2018). Theranostic gold-magnetite hybrid nanoparticles for MRI-guided radiosensitization. Nanotechnology.

[64] Li J., Zheng L., Cai H., Sun W., Shen M., Zhang G., Shi X. (2013). Facile one-pot synthesis of Fe_3_O_4_@Au composite nanoparticles for dual-mode MR/CT imaging applications. ACS Appl. Mater. Interfaces.

[65] Zhao H.Y., Liu S., He J., Pan C.C., Li H., Zhou Z.Y., Ding Y., Huo D., Hu Y. (2015). Synthesis and application of strawberry-like Fe_3_O_4_-Au nanoparticles as CT-MR dual-modality contrast agents in accurate detection of the progressive liver disease. Biomaterials.

[66] Wang W., Hao C., Sun M., Xu L., Xu C., Kuang H. (2018). Spiky Fe_3_O_4_@Au Supraparticles for Multimodal In Vivo Imaging. Adv. Funct. Mater..

[67] Ge Y., Zhong Y., Ji G., Lu Q., Dai X., Guo Z., Zhang P., Peng G., Zhang K., Li Y. (2018). Preparation and characterization of Fe_3_O_4_@Au-C225 composite targeted nanoparticles for MRI of human glioma. PLoS ONE.

[68] Smolensky E.D., Park H.-Y.E., Zhou Y., Rolla G.A., Marjańska M., Botta M., Pierre V.C. (2013). Scaling laws at the nanosize: The effect of particle size and shape on the magnetism and relaxivity of iron oxide nanoparticle contrast agents. J. Mater. Chem. B.

